# Lethal Suspected DICER1 Syndrome Spectrum in Neonate with CPAM Type IV/Type I PPB, Genitourinary Abnormalities, and CNS Embryonal Tumor with Multilayered Rosettes

**DOI:** 10.1177/10935266261449073

**Published:** 2026-05-30

**Authors:** Rachel Guest, John Van Arnam, Darryl Kinnear, Fouad El-Dana, Kelsey Hummel, Anthea Lafreniere, Eumenia Castro, Melissa Blessing

**Affiliations:** 1 Department of Pathology & Immunology, Baylor College of Medicine, Houston, TX, USA; 2 Independent Researcher, Houston, TX, USA; 3 Department of Pathology, Texas Children’s Hospital, Houston, TX, USA; 4 LTC Charles S. Kettles VA Medical Center, Ann Arbor, MI, USA; 5 The Hospital for Sick Children (SickKids) and the University of Toronto, Toronto, ON, Canada; 6 University of Wisconsin, Madison, WI, USA; 7 University of Iowa Health Care and Carver College of Medicine, Iowa City, IA, USA

**Keywords:** DICER1 syndrome, pleuropulmonary blastoma, embryonal tumor with multilayered rosettes, autopsy, ovarian anomaly, neonatal

## Abstract

DICER1 syndrome is a rare autosomal dominant cancer predisposition syndrome caused by germline mutations in the *DICER1* gene and several associated benign and malignant entities. This case describes a premature 8-day old neonate prenatally diagnosed with renal and cystic lung abnormalities who died from severe respiratory dysfunction. Autopsy additionally revealed ETMR and genitourinary abnormalities, leading to the diagnosis of probable DICER1 syndrome. While the family declined postmortem genetic testing, detailed postmortem examination led to important diagnoses capable of guiding clinical follow-up and future family planning.

## Introduction

DICER1 syndrome is a rare autosomal dominant cancer predisposition syndrome caused by germline mutations in the *DICER1* gene, while GLOW (Global developmental delay, lung cysts, overgrowth, and Wilms tumor) syndrome is associated with mosaic missense mutations within the RNAse IIIb domain of *DICER1.*^1^ The *DICER1* gene is located on chromosome 14q32.13. This gene codes for an RNase involved in siRNA/microRNA development and, in conjunction with mRNA, regulates up to 30% of genes integral to the process of making proteins.^2^ Abnormal RNA fragments result in dysmorphia and neoplasia. Benign entities associated with DICER1 syndrome include congenital pulmonary airway malformation (CPAM), multinodular hyperplasia, and cystic nephroma while malignant tumors linked to *DICER1* mutations include pleuropulmonary blastoma (PPB), sex cord stromal tumors, anaplastic sarcoma of the kidney, and embryonal tumor with multilayered rosettes (ETMR).^3,4^ While the exact prevalence of DICER1 syndrome is unknown and complicated by incomplete penetrance,^5^ carriers of the pathogenic *DICER1* variants enrolled in international registries have demonstrated an increased risk of neoplasia, with a 5-fold increased risk of neoplasia when compared to the general American population.^6,7^ We performed a postmortem examination of an 8-day-old premature female with prenatally diagnosed pulmonary and renal abnormalities, incidentally found to have an exceedingly rare primary CNS tumor. The constellation of autopsy findings strongly implicates *DICER1* mutation, indicating the importance of recognizing unifying features and rare entities.

## Case Report

An 8-day-old female neonate was born via cesarean section at 36-5/7 weeks gestational age to a 33-year-old G2P0 mother with known pelvic outlet obstruction. Maternal medical history was significant for chronic hypertension, epilepsy requiring Keppra, and third-trimester COVID-19 infection in a vaccinated patient. Twenty-week gestational age ultrasound showed no anatomical abnormalities. However, an ultrasound performed at 36-5/7 weeks gestation demonstrated diffuse bilateral lung echogenicity with significant left lung cystic changes displacing the heart and enlarged, echogenic kidneys.

The neonate was large for gestational age at birth, with slightly low set ears and macrocephaly. Chest x-ray confirmed lung cystic changes and showed worsening ground glass opacities and hyperinflation, concerning for CPAM and pulmonary hypertension. Cardiac ultrasound showed atrial septal defect versus patent foramen ovale with prominent patent ductus arteriosus. Renal ultrasound revealed enlarged kidneys without pyelectasis. The patient required ventilation from birth. Despite aggressive medical treatment including extracorporeal membrane oxygenation (ECMO), her lung cysts continued to expand with worsening hypoxia and multiorgan failure. Care was compassionately redirected and the patient died on the 8th day of life. Consent for complete postmortem examination was given. As this case was referred from an outside hospital, no further details concerning genetic consultations, family history, or possible familial genetic abnormalities could be obtained, other than declined neonatal genetic testing.

Autopsy external examination revealed dysmorphic features, including: flattened nasal bridge; irregular spacing of the bilateral index finger and thumb; pointed and distorted right ear; borderline elongated philtrum; and pectus excavatum with inverted, widely spaced nipples. Internal examination showed markedly cystic lungs, enlarged kidneys, and uterus didelphys with double-outlet vagina. Neuropathology demonstrated an incidental 1.8 cm white-tan papillary/cauliflower-like suprachiasmic mass and hippocampal malrotation.

Microscopy revealed diffuse bilateral cystic lung changes consistent with CPAM type 4 versus PPB type 1 spectrum (Figure 1(A)). The cyst walls showed increased cellularity with condensation of small to spindled cells creating a cambium-like layer, focally positive for desmin (Figure 1(B)). The cysts were lined by a single layer of plump cuboidal epithelium. Ovarian parenchyma displayed a disproportionately increased number of large cystic follicles for age, many containing multiple oocytes, consistent with disordered folliculogenesis (Figure 1(C)). The kidneys showed immature development with a discontinuous nephrogenic zone and no cyst formation (Figure 1(D)). Brain tumor histomorphology was diagnostic of Embryonal Tumor with Multilayered Rosettes (ETMR), CNS WHO Grade 4 (Figure 1(E) and (F)) involving the optic chiasm, third ventricle, and tectum.

**Figure 1.**
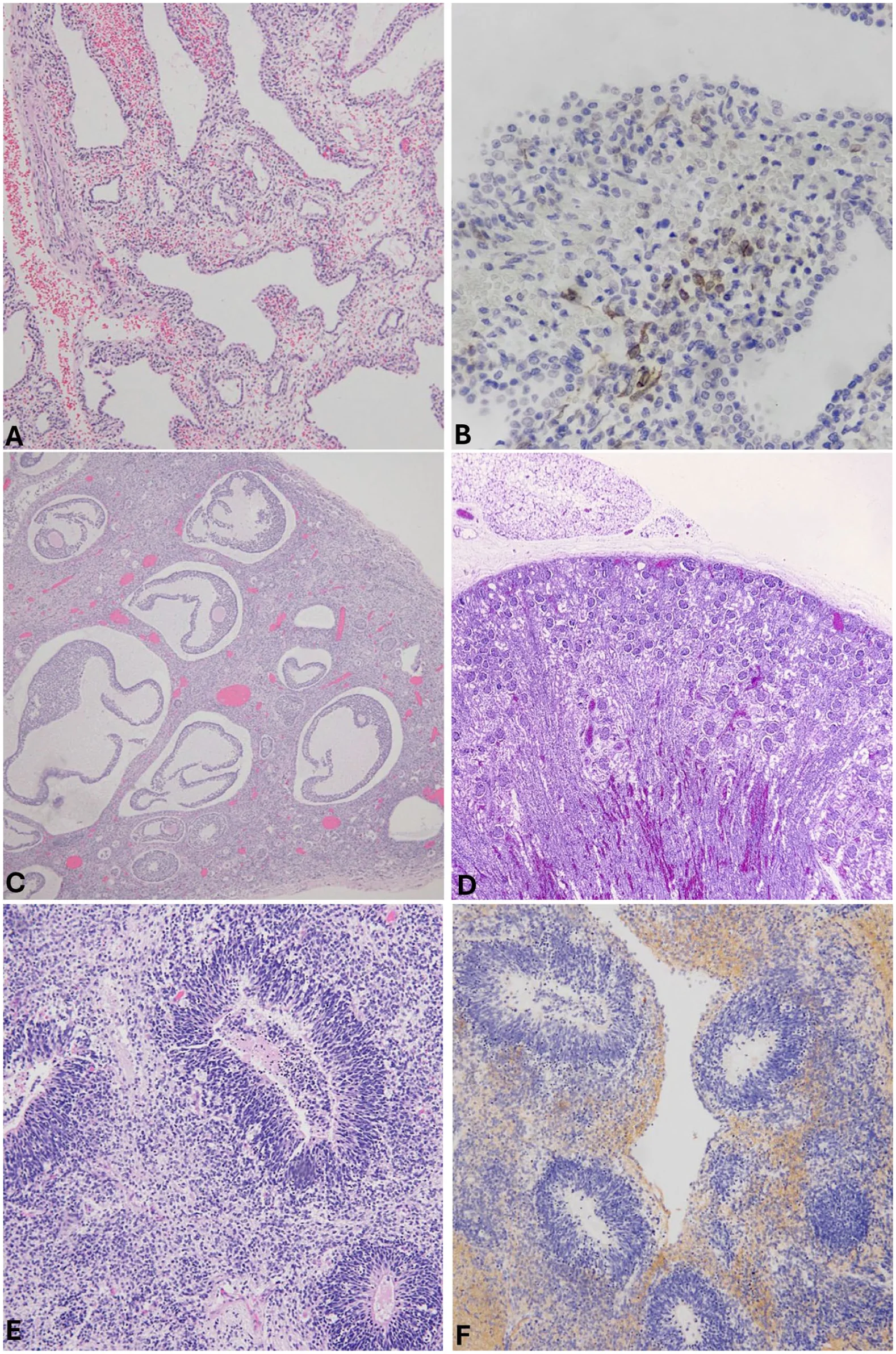
(A) Cystic lung parenchyma with increased cellularity with condensation of small to spindled cells (H&E; 100×) with (B) cambium-like layer focally desmin positivity (200×). (C) Ovarian parenchyma with disproportionately increased large cystic follicles for age, many containing multiple oocytes (H&E; 100×). (D) Immature kidneys with a discontinuous nephrogenic zone (H&E; 40×). (E) Brain tumor with layers of mitotically active neoplastic cells forming rosettes (H&E, 200×) and (F) neuropil highlighted by synaptophysin (200×).

Given the association between PPB, ETMR, and the unlikely contribution of antiepileptic medication,^8^ DICER1 syndrome was favored. Confirmatory genetic testing was declined by next-of-kin; however, parental genetic testing was strongly suggested in our autopsy examination report and in discussion with the clinical team.

## Discussion

The *DICER1* gene encodes over 1900 amino acids which are grouped into 7 domains: the helicase ½, ATP-binding, the helicase C-terminal, the Dicer dimerization, the PAZ, and the ribonuclease IIIa and IIIb domains, within which reported mutations include deletions, insertions, and other alterations.^9^ Tumorigenesis in most *DICER1*-associated tumors is best explained by the haploinsufficiency model, in which a germline mutation is followed by additional alterations that result in functional loss of this tumor suppressor gene.^10^ Most individuals with DICER1 syndrome inherit the mutation rather than acquiring it de novo.^11^

The most common pulmonary manifestation of *DICER1* mutations is PPB. This rare pediatric embryonal lung tumor arises from mesenchymal cell proliferation within cysts in the pulmonary airspaces.^12^ Grossly, this malignancy can present as cystic lesions (type 1), cystic lesions admixed with solid stromal components (type 2), or solid stromal expansion only (type 3).^13^ PPB type 1r is also characterized by cystic lesions but contains no malignant cells.^9^ The solid components of PPB can consist of an increased number of spindle cells, blastema cells, anaplastic rhabdomyoblasts, or immature cartilage.^13^ Pathogenic germline variants of the *DICER1* gene are seen in ~80% of children with this entity and somatic mutations are identified in most PPB specimens.^14^ Investigation into the development of PPB from CPAM type IV via somatic *DICER1* mutations is ongoing. CPAM type IV consists of pneumocyte-lined cysts without the rhabdomyoblasts or immature cartilage formation seen in PPB,^15^ making differentiation between CPAM type IV and PPB type 1r exceedingly difficult. Sporadic cases of CPAM with *DICER1* mutations have been reported. However, characterization of CPAM type IV remains uncertain, and large-scale studies examining its association with somatic *DICER1* mutations have not yet been conducted.

Several primary brain tumors, including Embryonal Tumor with Multilayered Rosettes (ETMR), an entity previously included in the CNS-primitive neuroectodermal tumor (CNS-PNET) group, are also associated with *DICER1* mutations.^3^ ETMR is an aggressive, CNS WHO Grade 4 brain tumor composed of dense proliferations of undifferentiated cells interspersed with fibrillary neuropil and multilayered rosettes with lumina.^3^ The immunohistochemical profile for this lesion is diffuse LIN28A positivity, p53 positivity in rosettes, GFAP and synaptophysin positivity in neuropil, and BAF47 negativity.^16^ While the majority of EMTRs are associated with microRNA dysregulation due to C19MC locus amplification with increased LIN28A expression, a subset of ETMRs instead show *DICER1* mutations in the ribonuclease III domain; these also display LIN28A positivity.^3^

DICER1 syndrome patients also develop benign and malignant tumors in the genitourinary system. Cystic nephroma, anaplastic sarcoma of the kidney, and nephroblastoma have all been associated with DICER1 syndrome,^5,17^ as have sex-cord stromal tumors and cervical embryonal rhabdomyosarcoma.^2,4^ Additionally, abnormal ovarian folliculogenesis has been described. Although occasional degenerative or cystic follicles are expected, numerous large cystic follicles, as seen in this case, are abnormal and have been associated with *DICER1* mutations in a prior mouse-model study, along with increased follicular degeneration and abnormal corpus luteum vascularization.^18^ Uterine structural abnormalities have also been associated with deletions in *DICER1*^18^; however, uterine didelphys has not been specifically reported and may represent a novel *DICER1* association. Non-neoplastic renal developmental abnormalities are also seen in *DICER1* mutant patients which may precede neoplastic transformation.^17^

While facial dysmorphism is not typical of classic DICER1 syndrome, it has been described in GLOW syndrome. An underlying GLOW syndrome warrants consideration given the facial dysmorphism observed in this case. However, GLOW syndrome remains incompletely defined,^9^ with no established association with neurologic tumors. In this setting of facial dysmorphism and presence of ETMR, the possibility of multiple *DICER1* alterations may be considered.

CPAM and EMTR are both rare entities. The incidence of CPAM in newborns is between 1:11 000 and 1:35 000.^15^ The incidence of EMTR has yet to be determined due to its rarity; however, the incidence of all CNS-PNET is estimated to be 1:700 000.^3^ The exceedingly small probability of independently developing a CPAM/PPB spectrum lung lesion and ETMR in conjunction with genitourinary abnormalities and dysmorphic features strongly supports inherited *DICER1* mutation, including syndrome. While the value of postmortem genetic testing is increasingly recognized,^19^ it may have limited availability or undesired by next-of-kin, as in this case. This report therefore highlights the utility of detailed postmortem examination and recognizing syndromic associations including the ever-evolving presentations of DICER1 syndrome.
